# 

**Published:** 1907-02-02

**Authors:** 


### THREE NEW BOOKS FOR NURSES

### *NOW READY.*

**A NEW AND IMPORTANT BOOK ON**

### SKIN DISEASES:

### THEIR NURSING AND PRACTICAL MANAGEMENT.

***2s. 6d. net.*** 2s. 9d. post free.

By G. NORMAN MEACHEN, M.D., B.S., M.R.C.P., M.R.C.S.

***2s. 6d. net.*** 2s. 9d. post free.

This is the only work on Skin Diseases written especially for Nurses. It deals with all the more common diseases and describes very fully usual forms of treatment. The work cannot fail to be of the utmost value to Nurses, and should prove a useful book for the household.

### A MANUAL FOR NURSES ON

### ABDOMINAL SURGERY.

***2s. 6d. net.*** 2s. 9d. post free.

By HAROLD BURROWS, M.B., F.R.C.S.

***2s. 6d. net.*** 2s. 9d. post free.

*CONTENTS*: Symptoms in Abdominal Disease—Peritonitis—Hernia—Affections of the Stomach—Affections of the Small Intestine—Intestinal Obstruction—Intussusception—Appendicitis—Affections of the Liver and Gall-bladder—Diseases of the Uterus and Appendages—Preparation for Abdominal Section—After-troubles and Complications of Abdominal Operations—Feeding.

**You have often been in need of a Book**

That would aid you in emergency. That would help you to recollect some point you had forgotten or about which you had a doubt.

That would prove of assistance in difficulty.

That would convey information clearly and concisely on Diseases; their Symptoms and Nursing Treatment.

### THE NURSE'S "ENQUIRE WITHIN"

is a Pocket Encyclopædia of valuable nursing information alphabetically arranged, and

**Fills a Want**

that has long been felt by Nurses everywhere. It is bound in limp cloth boards with rounded corners, and its size and weight will admit of its being carried conveniently in the apron pocket without injury thereto.

***2s. net; 2s. 3d. post free.***

**The Scientific Press, Ltd.,**

☞ *Catalogue of Nursing Manuals sent post free on application.* ☜

**Publishers of Nursing Text-Books, Charts, and Case-Books of all Descriptions.**

**28 & 29 SOUTHAMPTON STREET, STRAND, LONDON, W.C.**

